# Heterogeneity of Cognitive Profiles in Children and Adolescents with Mild Intellectual Disability (MID)

**DOI:** 10.3390/ijerph19127230

**Published:** 2022-06-13

**Authors:** Urszula Sajewicz-Radtke, Paweł Jurek, Michał Olech, Ariadna B. Łada-Maśko, Anna M. Jankowska, Bartosz M. Radtke

**Affiliations:** 1 Laboratory of Psychological and Pedagogical Tests, Czarnieckiego 5A, 80-239 Gdańsk, Poland; sajewicz-radtke@pracowniatestow.pl; 2 Institute of Psychology, University of Gdansk, Bażyńskiego 4, 80-952 Gdańsk, Poland; pawel.jurek@ug.edu.pl (P.J.); michal.olech@ug.edu.pl (M.O.); ariadna.lada@ug.edu.pl (A.B.Ł.-M.); anna.jankowska@ug.edu.pl (A.M.J.); 3 Department of Psychology, Medical University of Gdańsk, M. Skłodowskiej-Curie 3a, 80-210 Gdańsk, Poland

**Keywords:** Mild Intellectual Disability (MID), intelligence, psychological diagnosis, children, adolescents, cognitive functioning, Stanford-Binet 5

## Abstract

Mild Intellectual Disability (MID) is a neurodevelopmental disorder that begins in childhood and is characterized by limitations in intellectual functioning (IQ = 55–69) and adaptive behavior that manifests in everyday living. In addition to these specific criteria, clinical practice shows that the population of children with MID has heterogeneous deficits in cognitive functioning. Thus, the aim of this study was to identify groups of homogenous cognitive profiles within a heterogeneous population of students with MID. The cognitive profiles of 16,411 participants with Mild Intellectual Disability were assessed based on their performance on the Stanford–Binet Intelligence Scales–Fifth Edition. Prior to the assessment, participants were divided into three age groups corresponding to the levels of the Polish education system: (1) 7;00–9;11, (2) 10;00–14;11, and (3) 15;00–18;11 years old. Using cluster analysis, we identified three distinct cognitive profiles (clusters) in each age group. These clusters differed from each other within and between each age group. Distinguishing cognitive profiles among children and adolescents with MID is important both in the context of diagnosis as well as the development of research-based interventions for these students.

## 1. Introduction

Intellectual disability (ID) is a neurodevelopmental disorder that concerns approximately 3% of the general population and has an early onset; deficits are first recognized during infancy and childhood [1,2]. ID is characterized by intellectual (i.e., reasoning, problem solving, planning, abstract thinking, judgment, academic learning, and learning from experience) and adaptive functioning (i.e., independence and social responsibility) that are two or more standard deviations below the mean [3,4]. The severity of ID ranges from mild to profound and more than 75% of persons with ID have mild intellectual disability (MID) [4].

ID is an etiologically diverse condition [5,6], and the specific causes are often difficult to identify. Underlying biological, genetic, and neurological factors are most likely to be identified in persons with severe to profound ID, and they are responsible for even up to 75% of such cases [7,8]. Determining the etiology of MID is more difficult as it has a multifactorial origin consisting of a diverse set of factors, not limited solely to biology and genetics [2]. Environmental and psychosocial determinants (i.e., low socioeconomic status, low maternal education, malnutrition, and inadequate access to healthcare) are considered to be significant risk factors for MID [9,10,11].

Individually administered standardized tests of intellectual functioning and adaptive behavior as well as clinical assessment are the basis for ID diagnosis. The etiologic heterogeneity is reflected in clinical presentation as well as diagnostic evaluation results [1,2,12,13,14,15]. A high degree of variability in intellectual functioning and heterogeneity of cognitive profiles among persons with MID has been confirmed by only a few studies [16,17]. Most of the research approaches the population of people with MID as a unitary entity or as a single group with borderline intellectual disability (MBID) [1,2,18,19,20,21,22].

However, the results of several studies suggest that it is likely that the greater the level of intellectual disability, which is mainly caused by biological factors, the ‘‘flatter’’ (homogeneous) the cognitive profile is—with more severe delays in, for example, expressive language, vocal imitation, and development of socio-emotional abilities [23,24]. In the case of MID, where environmental factors play a significant role along with biological causes, it is expected that a certain level of variability will be present in their intellectual functioning and adaptability, as a result of the etiologic heterogeneity [14,19,24].

Fletcher et al. [14] distinguished four clusters within young children with MID according to their cognitive performance on 10 cognitive tasks that were designed to engage different processes (*relative size task, pointing task, phonological awareness task, oddity task, rhyming task, sequencing task, oppositional concepts task, taxonomic generation task, semantic information task,* and *word meaning task*). Results suggest, for example, that the first group of children performed better on the verbal tasks compared to other groups, whereas children in cluster 3 performed worse than children in clusters 1, 2, and 4 on phonological awareness tasks. However, their findings were limited due to the small sample size and exploratory nature of the research. In another study, Van der Molen et al. [17] indicated that children with MID in the three clusters that they identified have generally comparable IQ scores, but they presented differently in terms of memory skills. Children in cluster 1 showed average scores in verbal short-term memory and verbal working memory and also higher than average in visual short-term memory and visual working memory, whereas children from cluster 2 presented low scores on verbal working memory and visual short-term memory, as well as average scores in visual working memory. Lastly, cluster 3 children performed higher than average in verbal short-term memory, at the average level on verbal working memory, and low in visual short-term memory and working memory. On the other hand, Soenen [1] distinguished four subtypes of individuals with MID, considered more broadly than just through the prism of intellectual functioning. In clusters 1 and 3, she identified behaviors characteristic of personality disorders, whereas in clusters 2 and 4 she found developmental disorders. Furthermore, externalizing problems were identified in both clusters 1 and 4, and internalizing behavior problems were found in clusters 2 and 3.

Furthermore, research conducted by Schalke and colleagues [25] on the population of people with normal intellectual functioning showed the variability of the structure of intelligence over the years. Based on the above results, we decided to separate in our research three age groups in order to identify these differences in children and adolescents with mild intellectual disability. Educational stages in accordance with the Polish education system were adopted as the criterion for separating these groups.

The aforementioned studies indicate that the cognitive and adaptive functioning of children and adolescents with MID can be subdivided in different ways. However, there is a lack of research that addresses this issue in a systematic manner by analyzing the intellectual functioning profiles of children with MID in a large population, as we do in the present study. This is particularly because distinguishing cognitive profiles within the group of children and adolescents with MID is important for providing therapeutic help and educational adjustments for these students [2,12,17,23]. Therefore, the aim of this study is to investigate the heterogeneity of intellectual functioning of students with MID and to analyze whether distinctive cognitive profiles can be identified. The study is the first to use the Stanford–Binet Intelligence Scales–Fifth Edition (SB5), which provide an array of information regarding verbal and non-verbal intellectual functioning, to identify both strengths and weaknesses in the cognition of subgroups of students with ID.

The following hypotheses and research questions have been created on the basis of the presented theoretical background and clinical experience:

Research question 1: How many profiles of intellectual functioning can be distinguished in particular groups?

Research question 2: Will the same profiles of intellectual functioning be observed in each examined age group?

**Hypothesis** **1 (H1).** *We assume that students with MID constitute a heterogeneous group, differing qualitatively in terms of intellectual functioning profiles.*

**Hypothesis** **2 (H2).** *We assume that the studied age groups will have a profile indicating delayed speech development.*

## 2. Materials and Methods

### 2.1. Sample

This project was approved by the Ethics Board for Research Projects at the Institute of Psychology, University of Gdansk, Poland (decision no. 13/2022). Data were collected from *N* = 16,411 participants aged 7;00 to 18;11, whose results on the full intelligence (IQ) scale ranged between 55 and 69. All participants underwent complete intellectual disability assessment, including both functional level and intelligence testing by qualified diagnosticians from psychological and pedagogical counseling centers in Poland in the years 2019–2022. Information about the research was sent to psychological and pedagogical counseling centers all over Poland. Next, psychologists from the counseling centers that were interested in taking part in the research were trained in the research procedure. Most of the study participants were reported to the counseling centers by their parents or referred from educational institutions for the general assessment of cognitive functioning, most often due to experienced educational difficulties. During their visit in the counseling center, parents were informed by the psychologist that the counseling center takes part in the scientific research and then about the scope of the study and the data provided. After receiving the above information, the parents decided whether to consent to the child’s participation in the study or not. Eventually, participants came from all over Poland, and the structure of the study participants corresponds to the structure of the country’s population, taking into account regions and differentiation of the place of residence, as well as the mother’s education level. The written consent to participate in the study was collected from all parents, whose children took part in the study. No sensitive personal data were gathered. No payment was provided for participation in the study.

For each participant, a set of diagnostic results was collected. These included 10 subscales of the Stanford–Binet intelligence Scale–Fifth Edition [26]. The participants were divided into three age groups: from 7;00 to 9;11 (G1; *n* = 6092), from 10;00 to 14;11 (G2; *n* = 7109), and from 15;00 to 18;11 (G3; *n* = 3210). Table 1 presents details of sample composition for the three age groups.

### 2.2. Measures

Intelligence was measured using the Polish version of the Stanford–Binet intelligence Scale–Fifth Edition (SB5) [26]. The SB5 is widely used as an individual, specialized test for assessment of intelligence and cognitive abilities in the population and, in particular, in special needs groups. The test is based on the most current theory of intelligence [27]. The full IQ scale consists of 10 subscales (five nonverbal and five verbal) [26,28]. The nonverbal IQ scale is based on five nonverbal subscales referring to each of the five cognitive factors examined using the SB5 (Fluid reasoning, Knowledge, Quantitative reasoning, Visual-spatial processing, and Working memory). The verbal IQ scale is composed of five verbal subscales regarding each of the five factors. The reliability (measured using the omega factor) of the subscales ranges between 0.88 and 0.91 [28]. The average time taken to complete the full version of the SB5 test ranges between 45 and 90 min. For each of the subscales, a standardized score is calculated in the range of 1–19: this is the first level (of several) of the test results, which is calculated based on tables of norms and takes into account the age of the examined individual. Using the standardized scores ranging from 1 to 19 in the analysis allows for an easier interpretation of the results: the reference level is constant with an average score of 10 and a standard deviation of 3 [28]. Scores on this scale of 7 are one standard deviation below the mean or around the 16th percentile. Scores of 4 or less (two standard deviations below the mean) are 2nd percentile and below. The qualitative description categories were taken directly from the test manual [28,29,30].

### 2.3. Statistical Analysis

Cluster analysis was used in order to identify the intelligence profiles of the individuals in the Mild Intellectual Disability (MID) group. In the first step, in each of the age groups, the number of clusters was assessed. To this end, the NbClust function from the R NbClust package was used [31]. This function checks 30 different criteria to suggest the number of clusters in a dataset. All options between 3 and 12 were taken into account (distinguishing between just two clusters would be impractical, and in the opinion of the authors, it would not generate significant information about the characteristics of the diagnosed individuals; on the other hand, for more than 12 clusters, the number of clusters needing interpretation would be too big and not clinically useful). The maximum distance function was used in the model because we wanted to identify groups of profiles with regards to diagnosis, which behave similarly on each of the dimensions and not similarly in the sense of the sum of distances for each of the coordinates, as would be the case in classical Euclidean distance analysis.

The next step was to fit the cluster models for the number of clusters selected for each group. The k-means method with maximum metric was used for this. After fitting the model (randomized initial conditions were used in order to minimize the risk of incorrect fit due to a particular selection of initial conditions), we analyzed the profiles of the results assigned to the different clusters. All of the analyses were conducted in the R environment using RStudio software [32].

## 3. Results

**Figure 1:**
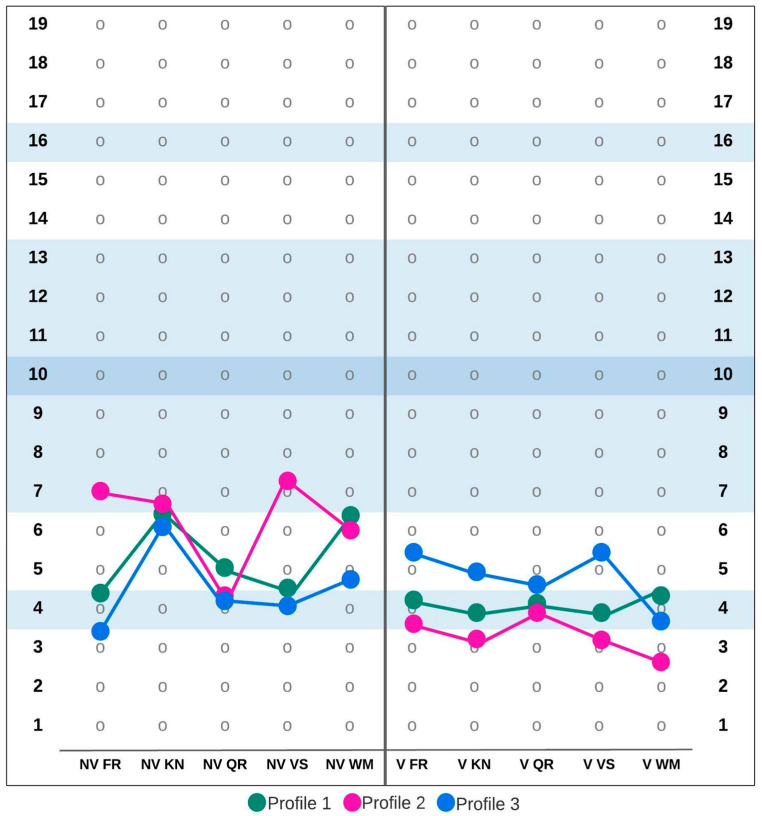
Mild intellectual disability cognitive profiles in the 7;00–9;11 age group. Abbreviations: NV FR—Nonverbal Fluid Reasoning, NV KN—Nonverbal Knowledge, NV QR—Nonverbal Quantitative Reasoning, NV VS—Nonverbal Visual-Spatial Processing, NV WM—Nonverbal Working Memory, V FR—Verbal Fluid Reasoning, V KN—Verbal Knowledge, V QR—Verbal Quantitative Reasoning, V VS—Verbal Visual-Spatial Processing, V WM—Verbal Working Memory.

**Figure 2:**
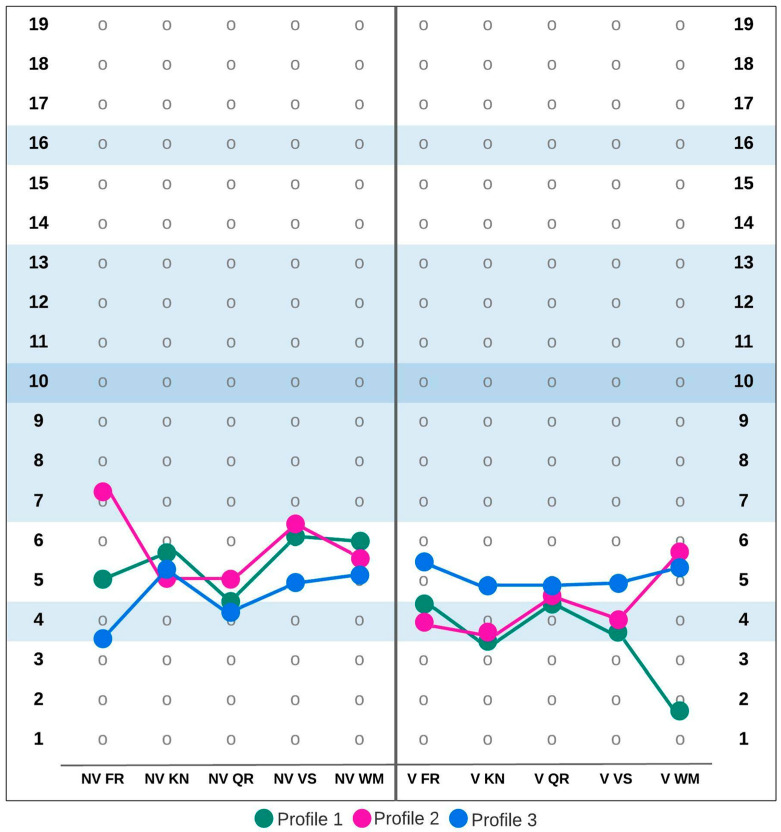
Mild intellectual disability cognitive profiles in the 10;00–14;11 age group. Abbreviations: NV FR—Nonverbal Fluid Reasoning, NV KN—Nonverbal Knowledge, NV QR—Nonverbal Quantitative Reasoning, NV VS—Nonverbal Visual-Spatial Processing, NV WM—Nonverbal Working Memory, V FR—Verbal Fluid Reasoning, V KN—Verbal Knowledge, V QR—Verbal Quantitative Reasoning, V VS—Verbal Visual-Spatial Processing, V WM—Verbal Working Memory.

**Figure 3:**
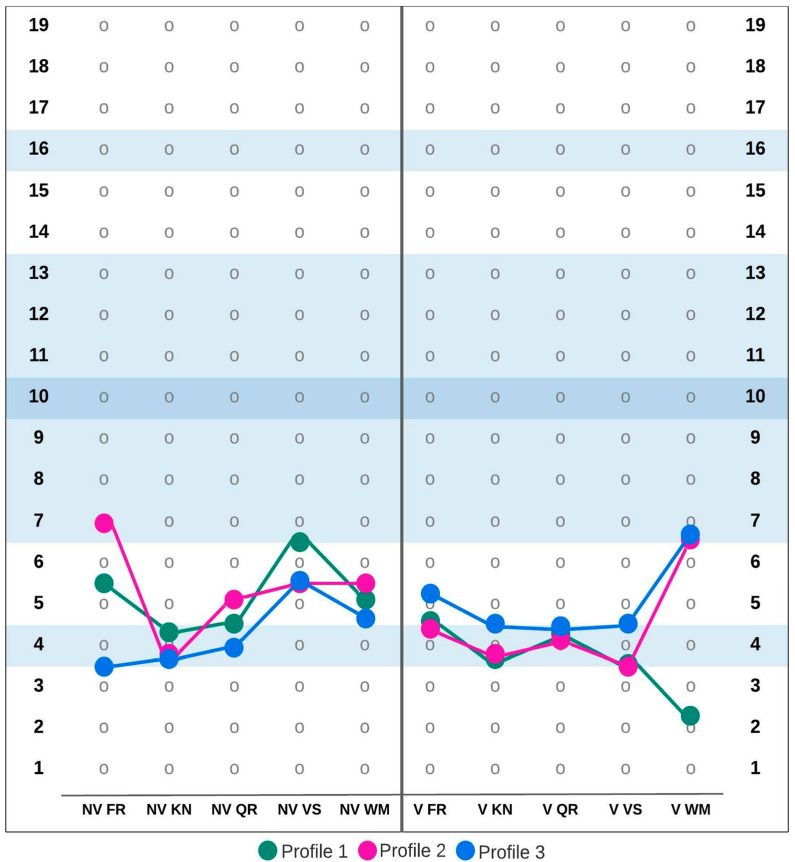
Mild intellectual disability cognitive profiles in the 15;00–18;11 age group. Abbreviations: NV FR—Nonverbal Fluid Reasoning, NV KN—Nonverbal Knowledge, NV QR—Nonverbal Quantitative Reasoning, NV VS—Nonverbal Visual-Spatial Processing, NV WM—Nonverbal Working Memory, V FR—Verbal Fluid Reasoning, V KN—Verbal Knowledge, V QR—Verbal Quantitative Reasoning, V VS—Verbal Visual-Spatial Processing, V WM—Verbal Working Memory.

Testing the optimal number of clusters revealed that each of the three age groups were best described by three clusters. This solution was supported by the largest number of tested criteria. Figure 1, Figure 2 and Figure 3 show the MID Cognitive Profiles for the different age groups based on cluster centers for ten SB5 subtests. Additionally, Table 2 summarizes the values of cluster centers in each of the distinguished groups.

In the 7;00–9;11 age group, 39% of participants (*n* = 2392) were assigned to profile 1. This profile was characterized by very low scores on the Nonverbal Fluid Reasoning and on all verbal scales as well as low scores on other scales. Profile 2 characterized 24% of the sample (*n* = 1480). This profile was characterized by results slightly below average on the Nonverbal Fluid Reasoning, Nonverbal Knowledge and Nonverbal Visual-Spatial Processing subscales but significant deficits on verbal subscales. The last profile in this age group (number 3) described 37% of participants (*n* = 2220). The third profile, in comparison to other profiles, was characterized by relatively higher scores (although still low) on the verbal subscales (with the exception of Verbal Working Memory, where the result was very low, as in the other groups) but relatively low results on nonverbal subscales.

In the 10;00–14;11 age group, profile 1 described 34% of the group (*n* = 2387). This profile was characterized by low scores on nonverbal subtests and very low scores on verbal subtests. Profile 2, which was observed in 38% of the participants in this age group (*n* = 2732), was characterized by results slightly below average on the Nonverbal Fluid Reasoning subscale. In the remaining nonverbal subtests, the results in this profile are low, similarly to the Verbal Working Memory subscale. There were deficits on other verbal subscales. The third profile in this age group described 28% of the participants (*n* = 1990) and was characterized by deficits in Nonverbal Fluid Reasoning and Nonverbal Quantitative Reasoning. In other subscales, the typical results for this profile could be categorized as “low”.

In the oldest group of participants (15;00–18;11) with MID, 38% of the group (*n* = 1223) were assigned to profile 1. This profile was generally characterized by low scores on nonverbal subscales and very low scores on verbal subscales. The second profile described 35% of participants (*n* = 1126). It was characterized by slightly lower than average scores on the Nonverbal Fluid Reasoning and Verbal Working Memory subscales and low or very low scores on the remaining subscales. The last profile in this age group, profile number 3, characterized 27% of participants (*n* = 861). Very low results on Nonverbal Fluid Reasoning, Nonverbal Knowledge, and Nonverbal Quantitative Reasoning subscales and low scores on the remaining subtests were typical for this subprofile.

## 4. Discussion

All three age groups of school-age children had a profile that indicated potential environmental and/or educational neglect (profile 2). In these groups, we observed lower mean scores for verbal competencies than for non-verbal ones, and the average results of the Knowledge scale were lower than the average results of the Fluid Reasoning scale. This conclusion is based on the assumption of the authors of the SB5 scale that verbal intelligence measured by Verbal IQ is one of the best predictors of academic success in the Western cultural sphere [19] and is assumed to be a better measure of crystallized intelligence [33,34,35,36]. The difference in profiles between Fluid Reasoning and Knowledge (in favor of the former) also supports the above conclusion. Fluid Reasoning is the ability to solve problems using inductive and deductive reasoning and to solve problem tasks that require understanding culturally independent relationships and dependencies. This factor is usually indicated as the best measure of fluid intelligence [27,34]. The Knowledge factor is, in turn, the best indicator of crystallized intelligence in SB5 [27,37]: it indicates the role of knowledge and skills acquired in the course of formal and informal education. This factor continues to grow to a greater extent than others throughout one’s life. This is the effect of accumulated experience and the impact of educational interactions [38]. Therefore, our findings indicate that many participants would potentially benefit from greater support within education settings, as well as more support for families to maximize opportunities and outcomes. Other studies also suggest that there may be a link between environmental and educational disadvantage and IQ outcomes. For instance, research conducted by Makharia and colleagues suggests that environmental factors such as place of residence, physical activity, family income, and parental education have impact on the IQ of the children [39]. Furthermore, Murtaza et al. [40] also associate father’s years of education, availability of educational materials, and responsiveness of the parent to the child with children’s intelligence. However, further exploration of the link between environmental and educational factors on child intelligence is necessary.

In groups of school-age children, the profile of probable coexistence of speech development disorders (primarily of understanding) with disability is visible. This conclusion is supported by the fact that in all analyzed groups on the verbal scale, the lowest score was Working Memory (profile 2 in G1, profiles 1 in G2 and G3), which is consistent with the delayed speech development profile from the SB5 Interpretation Manual [29]. The next two lowest average scores in these groups were Verbal Knowledge and Verbal Visual-Spatial Processing (both of these tests require a good understanding of verbal instructions and extensive verbal response). Moreover, these groups were characterized by a lower average of the verbal profile than the non-verbal one [29]. The highest average results were observed in non-verbal Visual-Spatial Processing; this test does not require the understanding of verbal instructions [27,36]. The person administering the test demonstrates how the answer should be given, and the answer does not require the use of language. In addition, in groups G1 and G3, the second highest, and in group G2, the fourth highest average scores (very close to the second and third highest Non-Verbal Working Memory and Non-Verbal Knowledge and relatively high in this profile), were Non-Verbal Fluid Reasoning. This test also requires neither understanding nor speech production [26,30].

Furthermore, in the two older age groups, G2 and G3, profile 1 may also indicate the occurrence of reading difficulties. Participants in profile 1 had generally low scores on non-verbal subtests and very low scores on verbal subtests. Moreover, very low results for Verbal Working Memory were observed, as well as generally lower results for Verbal Knowledge in relation to Non-Verbal Knowledge, and comparable results were observed in terms of quantitative reasoning for the Verbal and Non-Verbal areas. At this point, it would be worth considering the possibility of the coexistence of reading difficulties in the group of people with MID generally, as for now we cannot diagnose developmental learning disorder with impairment in reading in people with IQ scores below 70 [3].

Moreover, the observed differences in the structure of intelligence between individual age groups are consistent with the results of Schalke et al. [25], conducted among people in the intellectual norm. It can be assumed that the observed differences in the structure of intelligence between groups reflect different types of didactic and revalidation interactions at particular educational stages. It would be advisable to verify this thesis in further research.

## 5. Conclusions and Practical Implications

Based on different patterns of performance on SB5, we identified three types of homogenous cognitive profiles in the heterogeneous population of students with MID in each group of school-age children. This confirmed Hypothesis 1 and answered Research Question 1. However, only two of the profiles were found in all examined age groups (profiles indicating potential environmental and/or educational neglect and the coexistence of speech development disorders; Research question 2). Furthermore, Hypothesis 2 was also confirmed in our study–in all three groups of children and adolescents we distinguished a profile which may indicate delayed speech development to a much greater extent than in the other profiles. The decrease of these results is greater than expected in the group of people with MID.

Our research is, to our knowledge, the first to use SB5 to identify both strengths and weaknesses in cognition in a large sample of students with MID. The obtained results confirm the need for continuation of diagnostic interventions after diagnosis of intellectual disability, as already noted by clinicians. Frequently, the diagnosis of intellectual disability ends the diagnostic process, whereas our research shows that it is necessary to make an in-depth diagnosis, by using diagnostic tools that show the structure of the intellect, in order to design an adequate therapy which will be adjusted to the specific cognitive profile of a person, not only to the nosological classification. The profiles we have extracted indicate that it is extremely important to compare intellectual functioning in both the verbal and non-verbal areas, taking into account both these aspects of the examined intellectual functions when planning therapeutic interventions. Moreover, since intellectual disability is a neurodevelopmental disorder, the possible coexistence of intellectual disabilities with other neurodevelopmental disorders (e.g., ADHD) should be considered [4,12,24,41].

## 6. Limitations and Future Directions

The present study, despite valuable results for both scientists and practitioners, also has several limitations. First of all, unfortunately, we have no knowledge about the forms of therapy undertaken so far by the examined children and adolescents, as well as other support they have received so far. Furthermore, in future research, we would like to explore the possible role of family background in differentiating the cognitive profiles of children and adolescents from MID as well as look in depth at policy issues and the practical implications of clinical and educational settings. It would be also interesting to conduct similar analyzes in children and adolescents with MID from different countries and cultures and compare the results taking into account also cultural and policy issues on supporting people with MID.

## Figures and Tables

**Table 1:** Composition of the sample.

Variable	Age Group		
	G1 (7;00–9;11)	G2 (10;00–14;11)	G3 (15;00–18;11)
*Gender*			
Male	3788	4232	1837
Female	2224	2796	1339
Missing data	80	81	34
*Place of residence*			
Rural	2149	2527	1075
Urban	3698	4322	2033
Missing data	245	260	102
*Previous diagnosis*			
ADHD	28	30	6
Attention deficit disorder (ADD)	14	12	–
Speech impairment or SLI	82	37	11
Intellectual disability	611	1387	1068
Lower than average intelligence	748	1119	226
Neurological condition	32	42	19
Craniocerebral injury	2	6	2
Other	318	340	78
No diagnosis	4257	4136	1800

**Table 2:** Cluster centers (the arithmetic mean of all the observations belonging to the cluster) for 10 SB5 subtests on a standardized scale of 1–19.

	G1 (7;00–9;11)			*F*	G2 (10;00–14;11)			*F*	G3 (15;00–18;11)			*F*
Subtest	P1	P2	P3		P1	P2	P3		P1	P2	P3	
Nonverbal Fluid Reasoning	4.35	6.96	3.87	29.90 **	5.04	7.20	3.53	651.97 **	5.48	6.92	3.45	1649.21 **
Nonverbal Knowledge	6.49	6.76	6.13	35.08 **	5.73	5.07	5.31	181.72 **	4.21	3.87	3.79	0.82
Nonverbal Quantitative Reasoning	4.94	4.43	4.37	102.71 **	4.51	5.02	4.22	72.02 **	4.53	5.13	4.06	179.20 **
Nonverbal Visual-Spatial Processing	4.61	7.25	4.15	27.36 **	6.15	6.40	4.91	5.56 *	6.51	5.61	5.65	0.46
Nonverbal Working Memory	6.37	6.01	4.79	627.74 **	6.01	5.64	5.33	56.62 **	5.03	5.43	4.72	11.67 **
Verbal Fluid Reasoning	4.21	3.83	5.43	351.89 **	4.41	4.01	5.51	23.21 **	4.68	4.30	5.26	141.40 **
Verbal Knowledge	4.06	3.28	4.91	173.66 **	3.47	3.67	4.91	21.95 **	3.89	3.93	4.65	94.37 **
Verbal Quantitative Reasoning	4.18	3.90	4.67	50.46 **	4.26	4.65	4.92	66.29 **	4.23	4.15	4.46	30.14 **
Verbal Visual-Spatial Processing	4.06	3.23	5.44	402.60 **	3.86	4.02	5.04	14.25 **	3.68	3.56	4.64	235.93 **
Verbal Working Memory	4.30	2.75	3.82	63.55 **	1.81	5.79	5.29	3230.42 **	2.24	6.55	6.74	52.51 **

*Notes.* The *F*-value in an ANOVA testing the significance of the differences between cluster centers in each age group. * *p* < 0.05, ** *p* < 0.01.

## Data Availability

The presented data in this study are available from the corresponding author upon request.
